# “If everyone knew about this, how many lives could we save?”: Do drug suppliers have a role in reducing overdose risk?

**DOI:** 10.1016/j.dadr.2024.100250

**Published:** 2024-06-24

**Authors:** Bethany Hedden-Clayton, Jes Cochran, Jennifer J. Carroll, Alex H. Kral, Grant Victor, Erin Comartin, Bradley Ray

**Affiliations:** a Wayne State University, Center for Behavioral Health and Justice, 5447 Woodward Avenue, Detroit, MI 48202, USA; b The Never Alone Project, Indianapolis, IN, USA; c North Carolina State University, Department of Sociology and Anthropology, 10 Current Drive, Suite 334, Raleigh, NC 27606-8017, USA; d RTI International, 2750 Shattuck Avenue, Berkeley, CA, USA; e Rutgers University, School of Social Work, New Brunswick, NJ, USA; f Wayne State University, School of Social Work, 5447 Woodward Avenue, Detroit, MI 48202, USA; g RTI International, 3040 Cornwallis Rd, Research Triangle Park, NC 27709, USA

**Keywords:** Harm reduction, Peer training, People who supply drugs, People who use drugs

## Abstract

**Introduction:**

An unpredictable illicit drug supply is driving high levels of overdose death in North America. Prior research has demonstrated the importance of involving people who use drugs in harm reduction intervention design and implementation. The inclusion of people who supply drugs in these efforts has been scant. We explore this possibility by interviewing persons targeted by a harm reduction educational program designed specifically for people who supply drugs.

**Methods:**

In-person interviews with people who use drugs were conducted in 2022 in Indianapolis, Indiana. We conducted a thematic analysis of data from six interviews with people who were either primarily or secondarily trained through this harm reduction training for people who supply drugs,

**Results:**

Participants described a diverse array of harm reduction strategies, some gained through the targeted education program, which they regularly practiced as they consumed and/or supplied drugs to others. People who supply drugs were regularly identified as key actors capable of widely reducing risk across drug networks. Participants described being motivated by a moral imperative to protect community members, tying the previous loss of friends and loved ones to overdose to their commitments to the safety of others.

**Conclusion:**

This article contributes to the scholarship on the role of people who supply drugs in implementing harm reduction interventions and reducing overdose risk. Better enabling grassroots harm reduction organizations to provide people who supply drugs with harm reduction training and access to harm reduction resources may help to reduce drug-related harms.

## Highlights

• Many people who supply drugs welcome learning about harm reduction strategies.
• Harm reduction organizations can train drug use networks to promote safer use.
• People who supply drugs with a harm reduction philosophy are seen as reputable.
• People who supply drugs often share information about drug quality with customers.
• Suppliers and customers regularly exchange harm reduction strategies and tools.

## 1 Introduction

A major driver of overdose and other harms among people who use drugs in North America, is the variability and unpredictability of the unregulated drug supply (Jalal et al., 2018, Jones et al., 2018, Larnder et al., 2022, Scholl, 2019). While some are able to discern certain changes in the composition of the illicit drug supply (Duhart Clarke et al., 2022), many face general uncertainty about the content of the substances they use, rendering them unable to estimate dose or potency and increasing the likelihood of overdose, especially after a period of abstinence (Hayashi et al., 2018, McCrae et al., 2019, Nolan et al., 2019). In an illicit market that is increasingly characterized by novel adulterants (Singh et al., 2020), accessing a safe(r) supply has been increasingly acknowledged as an underutilized harm reduction strategy (Ivsins et al., 2020). While a well-regulated drug supply remains inaccessible, many people who use drugs rely on trusted individuals who supply drugs to support access to a more predictable supply, developing shared understandings of supply changes, and, when possible, communicating openly in order to reduce the risk of overdose (Carroll, 2021, Carroll et al., 2020, Measham, 2020).

Research conducted in North America suggests that people who supply drugs are often well-positioned to have an outsized impact on drug use-related health outcomes and behaviors, as their role in drug use networks can shape the risk and protective environment for people who use drugs (Carroll et al., 2017, Kolla and Strike, 2020, Palamar and Sönmez, 2022, Rhodes et al., 2019). The direct involvement of people who supply drugs—who may or may not also consume drugs (Potter, 2009)—in harm reduction activities has been most thoroughly addressed in studies detailing the utilization of drug checking services, many of which note that people who supply drugs regularly utilize these services to ensure the relative safety of products or to convey information to their customers about the drug products they provide (Bardwell et al., 2019, Long et al., 2020, Betsos et al., 2021). Apart from this emergent literature the potential of people who supply drugs to mitigate drug-related harms remains underexplored.

In this article, we analyze interview data from residents of Indianapolis, Indiana, all members of a social network that was targeted by a harm reduction educational program designed specifically for people who supply drugs at various levels of distribution. Below, we briefly describe the context of this community and program, then present interview data from persons who were trained by—or secondarily trained by a past participant of—this harm reduction educational program, with particular focus on the specific harm reduction strategies practiced and the moral valence motivating those strategies in the eyes of our participants. Our discussion highlights the potential for reducing drug-related harms when people who supply drugs are trained and empowered to practice harm reduction and present the potential impacts of policy support for those efforts.

## 2 Background

### 2.1 Marion County, Indiana

As of 2020, the U.S. state of Indiana was home to 6.7 million people (U.S. Census Bureau, 2021a), nearly one million of whom reside in the state capital, Indianapolis, located in Marion County (U.S. Census Bureau, 2021b). Marion County reported 2142 fatal and 9879 nonfatal overdoses between 01/01/2020—12/31/2022 (CDC, 2022). Indiana has implemented strict criminal consequences for drug possession and has classified the possession of a syringe without a prescription as a felony offense (IND.CODE ANN.§35–48–4–10; IND.CODE ANN.§16–42–19–18). Moreover, Indiana legislators enacted a drug-induced homicide law in 2018 (IND.CODE ANN.§35–42–1–1.5), allowing for the prosecution of a fatal overdose as equivalent to homicide (El-Sabawi et al., 2023, LAPPA, 2022). Indiana prosecutors have filed charges under this law (Hill, 2019) despite current evidence that these cases deter community members from calling 911 to report overdose emergencies (Carroll et al., 2021, Latimore and Bergstein, 2017, Ondocsin et al., 2020, Rouhani et al., 2021). Further, Indiana’s 911 Good Samaritan Law (IND.CODE ANN.§16–47–27–2) does not extend protection to the person experiencing an overdose and requires the person calling 911 to administer naloxone to receive protection under the law (LAPPA, 2022). This strict criminalization is the background against which drug use networks in Indianapolis have formed and evolved.

### 2.2 Harm reduction training for people who supply drugs in Indianapolis, Indiana

The Never Alone Project (NAP) is a peer-led harm reduction organization operating in Indianapolis. The “Community Harm Reduction Navigator” (CHRN), training was initially developed by NAP as a peer education program aimed at reducing overdose and disease transmission by teaching people who use drugs harm reduction strategies. During implementation, community members who supply drugs through wider drug use networks also enrolled, prompting NAP to ensure the training was relevant to this population. This included placing an increased focus on the particular needs, strengths, and opportunities represented by people who supply drugs, especially those who are, or whose drug networks include, persons who are Black, LGBTQIA+, and/or unhoused.

The CHRN curriculum consists of six semi-structured class sessions, each six-hours long, designed to be delivered once a week. Class sessions cover topics including: overdose prevention and overdose; safer drug use; legal rights education and community resource networking; drug culture and harm reduction practices; and infectious disease transmission and risk reduction. Participants were identified through social networks and word of mouth. The training is hands-on and includes role-playing so trainees can practice how they will explain and disseminate harm reduction supplies. At the time of data collection, two cohorts of 20 trainees had completed the training, each receiving a stipend of 1000USD and an additional 200USD to host two community training sessions with the aid of NAP-supplied harm reduction education materials.

## 3 Materials and methods

Data presented here were collected as part of a larger study which investigates the community impacts of drug interdiction (NIH RePORTER, 2021). Eligible participants for the parent study included people living in Indianapolis who reported recent use of opioids or stimulants obtained from within the local community. Participants were recruited through two local SSPs, one lead by the Marion County Public Health Department, the other by NAP. Data collection consisted of semi-structured interviews (BHC, JJC, GV, BR) conducted in the spring and summer of 2022. *A priori* domains covered in interviews included experiences in the local drug market, interactions with law enforcement, and strategies used to stay safe when using drugs. Interviews lasted between 25–75 min, were audio recorded and transcribed. We conducted a total of 33 interviews with this population and all were offered a 100USD gift card for their participation.

In this article, we focus our analysis on six of these 33 interviews, all of which were conducted with people who self-identified as having completed NAP’s training for people who supply drugs (n=3) or having been secondarily trained by others (n=3). The majority identified as members of the Black, Indigenous, People of Color community. These transcripts were thematically analyzed to identify common themes pertaining to harm reduction perceptions and practices. Using this method, all transcripts were iteratively coded (BHC). Emergent codes were regularly discussed by research team-members (BHC, BR, JC) and ultimately refined into a final codebook, which was applied to all transcripts. The findings presented here were produced in the final analysis. This protocol was reviewed and approved by the Institutional Review Board at Wayne State University.

## 4 Results

Three major themes emerged from our analysis: (1) the diverse ways in which participants promote and implement harm reduction strategies in their everyday lives; (2) how participants advocated for the adoption of harm reduction strategies within their wider social networks; and (3) participants’ self-described moral and social motivators for harm reduction practice.

### 4.1 The many harm reduction strategies adopted by people who supply drugs

Overall, participants described a variety of harm reduction strategies that had been integrated into their daily lives. In some cases, these were safer use techniques that participants engaged in the context of their own substance use such as testing drugs, using slowly, having access to naloxone and safer use supplies (e.g. sterile syringes), and using the buddy system or “spotting” each other (Spotting for People Who Use Drugs: What, When and How, 2021). Lauren, who reported consuming methamphetamine and opioids regularly, was at particularly high risk of fatal overdose, having experienced non-fatal overdose three times prior to her interview (Caudarella et al., 2016). She described learning about safer smoking practices through NAP’s harm reduction education program for people who supply drugs and expressed her excitement about sharing this information with others. After the training, Lauren switched her practice from injecting to snorting, because she felt she had more control over her dose and her high: “I used to shoot up and I stopped shooting up because I just felt like I didn't have much control over how much I was doing…when you shoot up, you know, you pretty much have to do all of it.” In this way, Lauren’s experience illustrates how targeted harm reduction education for suppliers can directly benefit the trainees, not just the persons to whom they supply drugs.

**Fig. 1:**
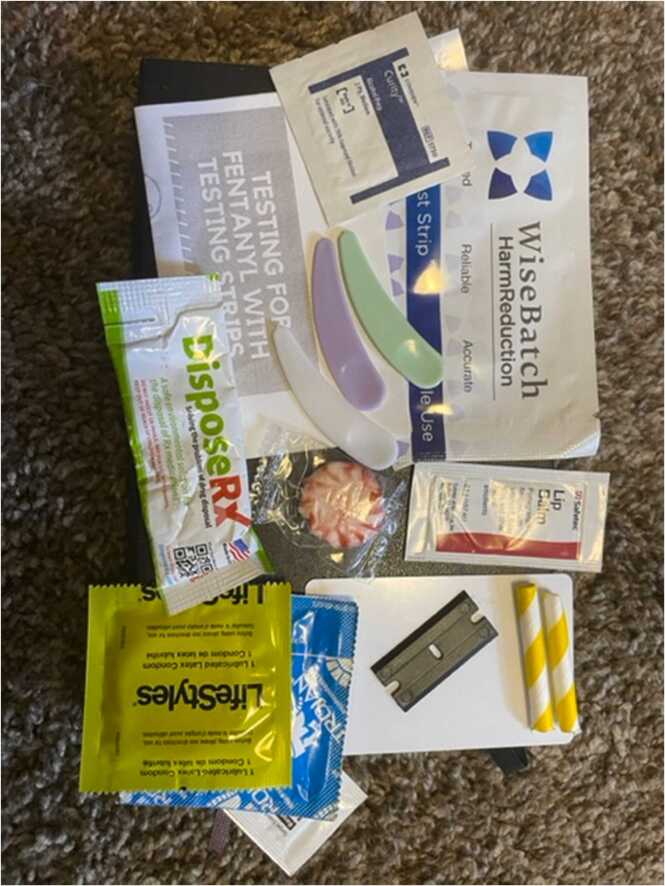
Safe snorting kit.

James, who self-identified as a person who supplies drugs and a regularly consumes cocaine and cannabis, was even more enthusiastic about adopting a wide spectrum of harm reduction practices and philosophies. He described himself as someone who wears the proverbial “white hat” of a community-based public health advocate in all aspects of his life. He noted, “I will go to a party, and everybody is turned up and doing their thing or whatever…but I’d be damned if you sitting over there, start foaming or you start going blue and I am not right there to go [*makes “spraying” noise imitating the sound of nasal naloxone*] and get your ass together.” When probed about his utilization of other harm reduction supplies, James also referenced his new, enthusiastic appreciation for safer snorting kits (see Fig. 1). “Snorting-fucking-kits, man. You never understand, until you are educated, how you could pass another disease if you don’t have separate straws.” James also described other safer use practices in which he often engaged as a person who uses drugs himself, such as using substances in well-lit places and telling friends when and where he was using. Pretending to talk to one of these friends, he says, “if I don’t call you back, bro…come kick my fucking door in.”

Some participants also emphasized the need for harm reduction education among populations they believed were at greater risk of overdose, often taking up the mantle of performing that education themselves. Zoë, who self-identified as a person who supplies drugs, expressed her concerns that younger persons faced especially high risk of overdose, stating, “a lot of my friends have been overdosing and I feel like nobody is standing up for what’s going on in the community. It’s a lot of teenagers and 20-year-olds that’s gone, and it keeps happening.” Zoë described grassroots efforts among friends to canvass neighborhoods with informational cards and resources, but described those efforts as futile. “The community looks at me like what’s she doing it for, you know, this is stupid. You’re not going to change nothing…I try my best, but I recently, I’m starting to give up…I feel like real dumb.” Despite her discouragement, Zoë persists in her belief that harm reduction strategies are essential for saving lives; however, her frustrations may indicate that advocacy efforts on her part and the part of others like her may be most effectively directed at targeted audiences of drug network members.

### 4.2 Dissemination of harm reduction strategies within drug use networks

Several participants described their advocacy to promote harm reduction strategies across their drug networks—including among other people who supply drugs—as highly successful. For instance, Nicole, who prefers MDMA, has advocated for the adoption of drug checking strategies among people who supply drugs in her social networks. Emphasizing the minimal quantity of product required to test one’s supply with fentanyl test strips (FTS), Nicole, described pitching drug checking efforts to people who supply drugs as follows: “Hey, you know, if you buy this jar [of product or pills], and you know that the jar all came from the same place, like sacrifice one [pill], bro, like, and just test it, you know what I’m saying?” When asked if people supplying drugs in her network often distribute FTS to their clients, Nicole replied, “Well, I can say this, I give them enough to make sure [they can], and they typically do ask for more.”

Nicole also recalled a recent conversation in which someone who supplies drugs remarked that the local stimulant supply might be contaminated with opioids. Nicole recalled, “I was just having a conversation with a homeboy…and he was like, ‘…just be careful because this is what I heard is going on.’ I was like…yes, let me go ahead and sit this summer out and try to make sure people have fentanyl testing strips.” Of note, Nicole’s anecdote illustrates well how improved customer knowledge about the local drug supply can shape drug use behavior: Nicole recounted abstaining from MDMA during this summer as a risk reduction strategy, opting to use cannabis and alcohol instead.

Joy also conveyed the benefit of knowing and communicating about contaminants in the drug supply with others. As a customer , Joy reported regularly testing the MDMA tablets she purchases with FTS. In this way, she determined that the product she purchased from a particular individual consistently tested negative for fentanyl. She shared this information with that person, and that person who supplies drugs reportedly tests his own product with FTS now. When asked how this person responded to her information about the consistently negative FTS results obtained from their product, Joy laughed and stated,

He said, “well, thank you. Thank you for telling me that my stuff wasn’t *that*. I appreciate it.” And [I said], “I appreciate *you*,” because, you know, everybody do[es] something to either have fun or to clear their mind but, you know, the things that I do, it’s just a party thing, sociable. I want to go out and have fun, but dang, I don’t want to go out and have fun and die.

Joy further emphasized the real risks that could be avoided by using FTS regularly by recounting an instance when she and a friend tested an MDMA tablet purchased from a different person who supplies drugs. She recalled, “We put it in water, and she tested it, and it came back positive for fentanyl.” When asked what she did with this information, Joy shared, “We told all our friends, like, don’t go to this person. Don’t buy those pills from them because they definitely are lacing their pills with fentanyl.” Relatedly, Lauren spoke about the importance of open communication with people who supply drugs. When asked how she gets information about the potency of her supply, Lauren stated, “I mean, just word of mouth, you know, the people that I get it from, they will tell me if it’s strong.” Joy went onto describe her community as “more family than just, you know, friends” and explained that her distribution of FTS to her community as an effort to “take care of [them]” because “it’s better to have this [FTS] than to not have this.”

### 4.3 Moral and social motivations for harm reduction practice

Participants consistently explained their motivation to learn about and adopt harm reduction practices as rooted in concern for the safety and wellness of their peers. James, for example, said, “I’ve been spreading the harm reduction message, and I believe in what we’ve got going on because, I’ve had to save some friends’ lives before. I’ve seen the actual impact of what we do.” He specifically mentioned two pivotal events that informed his claim. In the first, a friend of James’ overdosed on products he supplied. James reflected on how this event impacted his understanding of what is at stake, remarking, “It blew my fucking mind, and it changed my life forever, and I vowed that I would never let that happen to me again.” The second event was his own prior non-fatal overdose. He survived thanks to a community member who was carrying naloxone, a harm reduction tool to which he had not yet been introduced at that time. James presumed the overdose had been the result of fentanyl contamination in stimulants, in this case cocaine, that he had used. He described how he felt after snorting the product as, “I end up…throwing up for maybe about two or three hours, couldn't sit up straight, eyes rolling, you know what I mean. Really kind of, almost, damn near death even.”

Ryan, who consumes cocaine, also depicted his entry into harm reduction as motivated by grief and loss. “[I] got into harm reduction because, like, it has been hitting home really bad, losing a lot of people, like, so, it’s just been getting worse.” Ryan shared that in just a six-month period, five of his friends under the age of 30 died from overdose. Describing himself as relentless in spreading harm reduction education, Ryan depicted his actions as eventually annoying people as he frequently discusses and shares harm reduction strategies with most anyone. He stated, “I basically teach harm reduction to other people and just teach them how to use naloxone, spread the word, pass out safer drug use kits to my friends and family and just strangers. People get tired of me talking about it [*laughs*].” Similarly, Zoë found her way into harm reduction through loss. While wiping away tears, she shared, “we’re just losing a lot of important people that we need.”

In addition to the need to protect the safety of himself and his peers, James also articulated certain economic motivations that served as a secondary driver of his interest in harm reduction activities. He explained, “Ever since the game [supplying drugs] started there’s been rules. One of the most important ones is that you can’t make no money if everybody who pays you is dead.” The economic rationale to prevent harm to his clients was part of his messaging to others on the importance of harm reduction strategies. James described many people who supply drugs as amenable to this message, stating,

I look for people [who supply drugs] who care about themselves, care about the people they sell things too, and who care about their product…Anybody that doesn’t want to have this harm reduction info, then he’s not reputable because he doesn’t care about your safety and that’s just foolishness, you know? Like I said, who the hell wants everybody to die?

Importantly, James ended up his description of economic motivations by quickly returning to broader concerns of public health and community wellness. Indeed, James described himself as a health navigator and described his harm reduction advocacy as a core component of his health navigation mission: “As community activists or community health navigators, that’s our job. Even as just people, you know? If everybody knew about this [harm reduction] thing, how many lives could we save?”

## 5 Discussion

This article describes the extent of, dissemination of, and motivations for harm reduction practices among a sample of persons who were directly or secondarily trained through a harm reduction educational program for people who supply drugs in Indianapolis, Indiana. Interview data reveals that people who supply drugs are willing and able to adopt a wide variety of harm reduction practices; that suppliers are valued by many drug network members as sources of high-impact risk reduction activities; and that harm reduction practices pursued by people who consume and supply drugs are often motivated by the moral imperative to provide mutual aid as well as an intimate understanding of what is gained and lost depending on the availability of harm reduction tools.

These findings have immediate implications for policy and research. First, our data support the contention that initially inspired NAP’s specialized harm reduction education program targeting people who supply drugs: that people who supply drugs are uniquely positioned—*and uniquely motivated*—to take concrete steps that reduce the risks posed to a wide swath of community members by an unpredictable and unregulated drug supply. The majority of U.S.-based syringe service programs (SSPs) already recognize the importance of social and drug market networks in harm reduction efforts, with 65–68 % of such programs engaging in secondary distribution of supplies (Tookes et al., 2024). Indeed, according to emerging literature (Kolla and Strike, 2020) and the authors’ own experience working in and collaborating with SSPs across the U.S., people who supply drugs have long been hailed by harm reduction experts as key to the effective dissemination of harm reduction messaging and supplies across diverse communities of people who use drugs. In practical terms, this paper suggests that targeting suppliers in ways that explicitly acknowledge and/or appeal to their unique role in local drug use networks may be a profitable starting point for strengthening engagement efforts with this population. Potential engagement strategies for people who supply drugs to be included in trainings like NAP’s include providing lunch, snacks, cigarettes and lighters, cash stipends for attendance, as well as childcare and petcare at the training location. In addition, it is strongly recommended the training be held at an innocuous location by a harm reduction or mutual aid organization that is trusted and respected among local drug use networks. Future research should further explore additional strategies for engaging people who supply drugs in harm reduction interventions and the extent of the risk reduction impact that people who supply drugs can have on overdose and other risks in the community. Finally, local, state, and federal leaders should also consider how to support—or at the very least, how to avoid thwarting—these key stakeholders in saving lives in their own substance use prevention and response activities.

Second, emerging evidence suggests that drug criminalization worsens and even actively produces community overdose risk (Mital et al., 2020, Akiyama et al., 2021, Ray et al., 2023). This perverse relationship between criminalization and overdose is further demonstrated by the fact that vast majority of evidence-based overdose prevention strategies constitute context-specific exceptions from criminalization (Carroll et al., 2018). Yet criminal drug policy remains the norm, and even those statutory frameworks for implementing these evidence-based carve-outs of criminal policy, such as the legalization of drug checking tools and Good Samaritan Laws, vary substantially from state to state (LAPPA, 2020, LAPPA, 2022, Reed et al., 2022). Our data suggests another way in which criminalization could exacerbate community overdose risk: when people who supply drugs are removed from these social networks through incarceration, displacement, or death, their knowledge—rooted in the embodiment of harm reduction practices and community-based practices of ethics and care (Betsos et al., 2021; Kolla and Strike, 2020; Wagner et al., 2014)—and the harm reduction practices that knowledge supports are also removed.

These findings should be interpreted with certain limitations in mind. First, our sample size is small (n=6) and not necessarily representative of other social networks, especially where those social networks are separated by differences in race, class, geographic location, or other major socio-cultural factors. Larger, representative studies may identify other key trends that were not detected here. Second, interviews in the parent study were not designed to elicit views of or experiences in NAP’s harm reduction education program. Our analysis constitutes a post hoc interpretation of data collected from persons known to have participated in this program.

## 6 Conclusion

This article demonstrates the potential for the role of people who use, sell, or share drugs to be expanded to include the distribution of evidence-based harm reduction interventions to reduce overdose and other drug-related harms. Our findings highlight the importance of two-way information sharing in supplier-customer relationships, in which trust may be critical. Building on that trust expands what types of social relations between drug use networks are possible so that we can mitigate the harms in a drug market marked by deadly variability. These efforts could be achieved by supporting people who use, sell, or share drugs in leading and developing these efforts.

## Funding

This work was supported by the 10.13039/100000030Centers for Disease Control and Prevention (Award# 1R01CE003362-01).

## CRediT authorship contribution statement

**Erin Comartin:** Writing – review & editing, Supervision, Project administration. **Bradley Ray:** Writing – review & editing, Supervision, Project administration, Investigation, Funding acquisition, Conceptualization. **Grant Victor:** Writing – review & editing, Investigation. **Jennifer J. Carroll:** Writing – review & editing, Investigation. **Alex H. Kral:** Writing – review & editing. **Bethany Hedden-Clayton:** Writing – review & editing, Writing – original draft, Project administration, Investigation. **Jes Cochran:** Resources.

## Declaration of Competing Interest

No conflict declared.
